# How to Assess the External Validity and Model Validity of Therapeutic Trials: A Conceptual Approach to Systematic Review Methodology 

**DOI:** 10.1155/2014/694804

**Published:** 2014-01-19

**Authors:** Raheleh Khorsan, Cindy Crawford

**Affiliations:** ^1^Military Medical Research, Samueli Institute, 2101 East Coast Highway, Suite 300, Corona Del Mar, CA 92625, USA; ^2^Department of Planning, Policy, and Design, School of Social Ecology, University of California Irvine, Irvine, CA 92697-7075, USA; ^3^Samueli Institute, 1737 King Street, Alexandria, VA 22314, USA

## Abstract

*Background*. Evidence rankings do not consider equally internal (IV), external (EV), and model validity (MV) for clinical studies including complementary and alternative medicine/integrative medicine (CAM/IM) research. This paper describe this model and offers an EV assessment tool (EVAT©) for weighing studies according to EV and MV in addition to IV. *Methods*. An abbreviated systematic review methodology was employed to search, assemble, and evaluate the literature that has been published on EV/MV criteria. Standard databases were searched for keywords relating to EV, MV, and bias-scoring from inception to Jan 2013. Tools identified and concepts described were pooled to assemble a robust tool for evaluating these quality criteria. *Results*. This study assembled a streamlined, objective tool to incorporate for the evaluation of quality of EV/MV research that is more sensitive to CAM/IM research. *Conclusion.* Improved reporting on EV can help produce and provide information that will help guide policy makers, public health researchers, and other scientists in their selection, development, and improvement in their research-tested intervention. Overall, clinical studies with high EV have the potential to provide the most useful information about “real-world” consequences of health interventions. It is hoped that this novel tool which considers IV, EV, and MV on equal footing will better guide clinical decision making.

## 1. Introduction 

External validity and model validity of study results are important issues from a clinical point of view. From a methodological point of view, however, it appears that the concept of external validity and model validity is far more complex than it first seems. As we begin to enter a time realizing the need for more mixed-methods designs and comparative effectiveness studies to be executed for making better informed health care decisions, the need for attention to some of these issues in evaluating study quality is imperative.

Systematic reviews in health care generally assess the quality of experimental randomized clinical controlled trials (RCTs). These systematic reviews are designed to identify and appraise methodological bias in reports of RCTs and synthesize the research evidence relevant to a specific research question. Therefore, the results of systematic reviews are often applied for policy making in health care and often regarded as the strongest form of research evidence, becoming a crucial component in helping make accurate decisions about clinical care. Nevertheless, the assessment of study quality in most health care systematic reviews is based on results weighted heavily according to internal validity.

In 1995, Moher and colleagues identified 25 scales and 9 checklists that had been used to assess bias of randomized trials [1, 2]. More recently, in 2008, Olivo and colleagues identified 21 scales that had been used to assess bias of randomized trials [3]. The majority of these quality assessment scales/checklists place primary emphasis on internal validity and assess only blinding, randomization also known as random allocation sequence, allocation concealment, and withdrawals and dropouts. Note that, while the majority of these tools are scales, which become aggregated scores in systematic reviews, organizations such as the Cochrane Collaboration recommend that systematic reviews avoid aggregation. In fact, according to the Cochrane Collaboration, the difficulty in assessing bias using scales and checklists is incomplete reporting by studies and subjectivity of assigning weights to scale categories. That is, is randomization more or less important when compared to blinding? In addition, often, bias scales place greater importance in reporting methods rather than appropriately conducting research methodology [4].

It is possible that such scales limit the quality analysis of the majority of systematic reviews, especially when making clinical decisions about health care and how the information applies to real-world situations, including RCTs and nonrandomized studies.

Therefore, systematic review tools such as the Cochrane Collaboration's tool Risk of Bias [4], the Consolidated Standards of Reporting Trials (CONSORT) [5], the Grading of Recommendation, Assessment, Development, and Evaluation (GRADE) approach [6], and the Scottish Intercollegiate Guidelines Network (SIGN) [7] are preferable when assessing bias in research studies and systematic reviews.

We believe that study quality is a multidimensional concept. This review discusses the concept of study quality and how it relates to internal, external, and model validity. We also outline the methods used to assess quality and introduce a tool developed by the authors called the external validity assessment tool (EVAT©), building upon what is already found in the literature in this area, to be more robust and streamlined, which can be used to assess external and model validity in clinical trials, along with internal validity criteria.

### 1.1. Concept of Study Quality

What is validity? Validity is the degree to which a result from a study is likely to be true and free from bias [8]. Interpretation of findings from a study depends on both internal and external validity. Generally in experimental clinical trials the effect of the intervention is measured based on outcomes estimated based on the persons who are enrolled in that trial. Those individuals who are enrolled are referred to as the “study population” or “study sample.” Internal validity signifies whether the study results and conclusions are valid for the study population. Therefore, it can be concluded that a study possess internal validity if a causal inference (also known as reciprocal relationship) can be properly demonstrated using three criteria: (1) the cause precedes the effect in time (temporal precedence), (2) the cause and the effect are related (covariation), and (3) there are no plausible alternative explanation for the effect other than the cause (nonspuriousness) [9]. Hence, experimental research attempts to accomplish the above criteria by (1) manipulating the presumed cause and observing an outcome afterward (treatment effect); (2) observing whether variation in the cause is related to variation in the effect; and (3) finally, using methods during the experiment to reduce the plausibility of other explanations for the effect. Therefore, true experiments are known as the “gold standard” in causal research and systematic reviews then become the highest form of evidence describing the total effect of experimental research when combining those true experiments together. However, it is difficult to meet the criteria for validity without defining (A) inferences about whether the causal relationship holds over variation in persons and measurement variables (external validity) and (B) the particular treatments and settings in which data are collected (model validity).

It is believed that internal validity is a prerequisite for the external validity and efficacy and effectiveness exist on a continuum [10, 11]. “Study results that deviate from the true effect due to systematic error lack the basis for generalizability” [11]. Without generalizability the true therapeutic effect of clinical trials cannot be assessed. As Dekkers and colleagues [11] state, “from a clinician's point of view the generalizability of study results is of paramount importance. According to the CONSORT statement external validity should be addressed in reporting randomized clinical trials (RCTs)” [11, 12]. With that said, it is staggering how often external validity is neglected in the methodological considerations of health care research [11, 13, 14]. Dekkers et al. also argue that there are two reasons why most clinical trials neglect external validity: (1) most clinical trials focus their outcome assessment based on the specific, narrow and “ideal” setting, and ignore the question to whether the intervention has an effect when applied in general practice or “routine clinical practice” and (2) researchers underestimate the complexity of external validity and often conceptualize external validity to a “deceptively simple description” of those persons not included in the study [11]. Therefore it is important to make a distinction between research finding of efficacy and effectiveness of an intervention for health care providers, policy makers, and other stakeholders. “Efficacy trials (explanatory trials or [RCTs]) determine whether an intervention produces the expected result under ideal circumstances. Effectiveness trials (pragmatic trials) measure the degree of beneficial effect under ‘‘real-world” clinical settings [15]. Hence, hypotheses and study designs of an effectiveness trial are formulated based on conditions of routine clinical practice and on outcomes essential for clinical decisions. Clinicians and policy makers often distinguish between the efficacy and the effectiveness of an intervention” [10]. In 2006, Gartlehner and colleagues reported that systematic reviews, including meta-analyses, were including bias assessment for efficacy trials and often ignoring assessment of effectiveness trials. They proposed and tested a tool that can assist researchers and those producing systematic reviews, as well as clinicians who are interested in the generalizability of study results, to distinguish more readily and more consistently between efficacy and effectiveness studies. This tool tested the primary factors in generalizability including patient baseline characteristics (e.g., gender, age, severity of the disease, and racial groups), geographic settings (urban versus rural) and health care systems and health outcomes, study duration and clinically relevant treatment modalities, assessment of adverse events, adequate sample size to assess a minimally important difference from a patient perspective, and intention-to-treat analysis. They state, “Random allocation, allocation concealment, and blinding negate these factors, thereby increasing internal validity on the one hand and decreasing external validity on the other. Therefore, to some extent, the operational definition of ‘‘effectiveness trial” delineates the necessary trade-offs with internal validity. An ideal definition would balance this equilibrium at a point at which satisfactory internal validity accompanies a high degree of generalizability” [10].

The following literature review will discuss how internal validity and external validity are equally important in deciding the effectiveness of treatment (both efficacy trials and effectiveness trials) for a specific condition or population, and by neglecting external validity from the systematic review quality assessment process researchers significantly reduce the overall quality of systematic review results and interpretations for translation of the evidence into practice.

### 1.2. What Is External Validity?

According to the classic study by Cook and Campbell, external validity is the inference of the causal relationships that can be generalized to different measures, persons, settings, and times [16, 17].

External validity concerns the generalizability of study; that is, how likely is it that the observed effects would occur outside the study? For this paper, we separate external validity into two separate terms: (A) external validity as the results to persons other than the original study sample (the population of patients to whom the results should be generalizable to the target population) and (B) model validity as the generalization of results from the situation constructed by an experimenter to real-life situations or settings (generalizability across situations or settings, that is, practitioners, staff, facilities, context, treatment regimens, and outcomes). An umbrella term “external validity” including model validity as the subset is traditionally used to describe *everything in a bucket* that falls outside internal validity [11]. External validity as defined by this paper is sometimes referred to as *population validity* and model validity is sometimes referred to as *ecological validity*.

But why is external validity important? And why should we measure it? In patient treatment, clinicians often are confronted with the question “what treatment plan is best for this patient?” [18]. In health care research, a similar question is often asked, “what is the most effective intervention for this patient population?” As stated previously, both clinicians and researchers turn to the evidence provided by RCTs analyzed in systematic reviews to give a quick approximation of the quality of evidence regarding that given intervention. Again, “these ranking systems define strength of evidence primarily on the basis of the internal validity. Dimensions of external validity are generally viewed as second-order issues to be resolved in the process of applying the evidence” [18]. Therefore, in health care and public health research internal validity seems to be the priority today [19]. However, as research becomes more applied and pragmatic, we see a trend towards emphasizing and strengthening external validity in clinical studies [16]. For example, it is important to know not only that a health care intervention or program works under ideal conditions (i.e., efficacy) but also that it is likely to be effective in other settings when deployed in the field in routine circumstances and with other populations (i.e., effectiveness).

Model validity, which we are calling a subset to external validity, also known as ecological validity goes beyond the patient eligibility criteria and moves to the conceptual model involving etiology, setting, and practice characteristics. In fact, often, the definition of external validity includes study generalization to population and setting. Here model validity takes into account (1) patient and provider preferences and knowledge; (2) skills, training, or accreditation of provider or treatment center(s); and (3) feasibility of treatment center(s) or study site to represent “real-life” environments.

Model validity is especially important in the evaluation of studies not only when testing the efficacy but also more importantly when combining it with effectiveness of CAM/IM studies. Jonas and Linde [20] discuss the differences between conventional and CAM and the conceptual systems being investigated. They state, “In the West, clinical research in conventional medicine relies on common assumption etiology, diagnosis, and pathophysiology, so there is no need to evaluate whether research methods have violated these basic assumptions. Many complementary medicine practices, however, come from systems of medicine developed outside these standard assumptions of Western medicine. Therefore, it is important that the researcher considers the interaction of the research methods with the conceptual model being investigated. They should determine how well a study has incorporated a CAM system's conceptual model into the investigation” [20]. As the field moves toward the acceptance of more mixed-methods designs, it will be key to evaluate quality in a systematic review not only according to internal validity (used primarily in efficacy trials) but also according to external validity as well to ensure that studies are addressing “real-life” applicability of the results being generated.

The objective of the present review is to evaluate the current evidence base available in the literature regarding external validity and model validity and develop and apply this assessment tool to measure external validity and model validity in randomized and non-randomized research trials that can be used with standardly accepted internal validity tools that will become more sensitive to areas where the reductionistic model does not fit (i.e., more pragmatic RCT and comparative effectiveness research (CER) designs focused more on effectiveness trials).

### 1.3. Literature Review for the Development of Tool

#### 1.3.1. Generalizability: Study Population versus Source Population

Because external validity depends on a source population (a.k.a. target population), the first step in the assessment of the external validity is to define this *source *population [11]. The source population is identified as those individuals in the general population who, on the basis of inclusion and exclusion defined domains, could be participants in the research study. For example, the source population can be population of all male patients with the treatment of heart failure with spironolactone admitted to hospitals in the Northeastern part of the United States. The* study *population (a.k.a. study sample or study subjects) are individuals randomly selected as a subset of the individuals from the larger source population. This is referred to as simple random sampling. For example, the study population now consists of male patients between the ages of 18 and 45 admitted to four hospitals in the North Eastern part of the United States who have a history of heart disease being treated with spironolactone for heart failure between the dates Jan 1, 2011 and Jan 1, 2012. This technique may look simple; however Dekkers and colleagues (2009) [11] describe it to be quite complicated:

“The problem is that in clinical practice different doctors may want to apply the same research evidence to different target populations. For instance, suppose a study exists on the effect of antihypertensive drugs in patients between ages 45 and 74 years, with diastolic dysfunction but without severe co-morbidity. There are several possibilities to define target populations for this specific study. One doctor might strictly want to generalize to persons in the age bracket 45–74 years. However, another may wish to apply the results to all adult hypertensive patients with diastolic dysfunction without severe co-morbidity; referring to hypertensive patients < 45 years as well as >74 years.

Indeed, if patients > 74 years were excluded from a study, should its external validity be restricted to patients below this age limit? Is there any reason to believe that the effects of the therapeutic intervention are not generalizable to 76-year-old patients? Or to those who are 77 years old? Likewise, would the results not be generalizable to a 40-year old? But where should this extension of generalizability stop? Next, the severity of co-morbidity might be perceived in different ways. Should uncomplicated diabetes mellitus, treated with oral medication, considered to be a severe co-morbidity? And what about diabetes treated with insulin? It becomes clear that there is no single commonly agreed predefined target population for a given study. The question on generalizability must be pondered for various target populations, that is, different types of patients, and the external validity should be assessed for each (pp3) [11]”.

In other words, “the sample studied in an RCT should be strictly representative, ideally by being drawn at random from the target population of interest, so that statistical inferences can be made.” A limitation to community-based programs especially family-based prevention program is motivation to participate. Participation, recruitment, and retention rates are highly variable. They argue that, “While a simple analysis of socio-demographic characteristics in participants versus non-participants is informative, we argue that it is not sufficient to rule in or out the effects of non-participation” [21]. Indeed, sample attrition is also viewed as a problem to generalizability. A separate attrition analysis is needed to explore whether there are any differences between dropouts and nondropouts in any risk factors or mediating variables.

Fernandez-Hermida and colleagues (2012) [21] also distinguish generalizability from applicability. They assessed three domains of external validity characteristics (generalizability, applicability, and predictability (GAP)) for 29 randomized trials that evaluated effects of universal family-based prevention programs on alcohol misuse in young people. Their study defines applicability as the “extent to which a preventive intervention, with demonstrated outcomes, can be judged effectively for relevance to a different setting and/or to a different population group” [21]. Lastly, they add predictability to their assessment. Their definition of predictability involves the extent to which study outcome measures relate to meaningful health or social outcomes (i.e., injury, morbidity, mortality, quality of life, and educational and economic achievements).

Lastly, they formalize external validity under deliberately narrow definitions. But when assessing external validity the authors reported that “information needed for adequate assessment of external validity was poorly reported across the studies” [21]. Fernandez-Hermida et al. [21] point out that:

“Variables such as gender or ethnicity of the child, educational level of the parents or family income level are used repeatedly, but there is no explanation of how these variables might affect the result of the intervention. If self-selection is present in the recruitment of a sample or its retention in both control and experimental groups, external validity, specifically the degree of generalizability of study results, may be limited (p1575) [21].”

Therefore, according to the above definition of *source* population, this can be problematic in conceptualization especially in RCTs that have been strictly defined by the eligibility criteria because a target population that perfectly fits the eligibility criteria will, by definition, still differ from the original study population with respect to geographical, ethnical, and temporal conditions. Each of these differences may affect each outcome of interest [11]. It is possible that a well-defined formalization of external validity can help facilitate its assessment.

In response to these problems, our recent efforts have been directed toward the development of a methodological checklist for external validity and model validity in health care RCTs, especially in the field of complementary and alternative medicine/integrative health care (also known as integrative medicine).

#### 1.3.2. Selection Bias versus Allocation Bias

There are several threats to external validity that can compromise our confidence in stating whether the study's results are applicable to other groups. One major threat is selection bias which is the effect of some selection factor of intact groups interacting with the experimental treatment that would not be the case if the groups were randomly selected. According to Shadish et al. (2002) [9]:

“The issue is most acute when the sample was randomly selected from the population. Consider why sampling statisticians are so keen to promote random sampling for representing a well-designed universe. Such sampling ensures that the sample and population distributions are identical on all measured and unmeasured variables within the limits of sampling error. Notice that this includes the population label (whether more or less accurate), which random sampling guarantees also applies to the sample. Key to the usefulness of random sampling is having a well bounded population from which to sample, a requirement in sampling theory and something often obvious in practice. Given that many well bounded populations are also well labeled, random sampling then guarantees that a valid population label can equally and validly be applied to the sample. With purposive sample selection, this elegant rationale cannot be used, whether or not the population label is known [9].”

**Figure 1:**
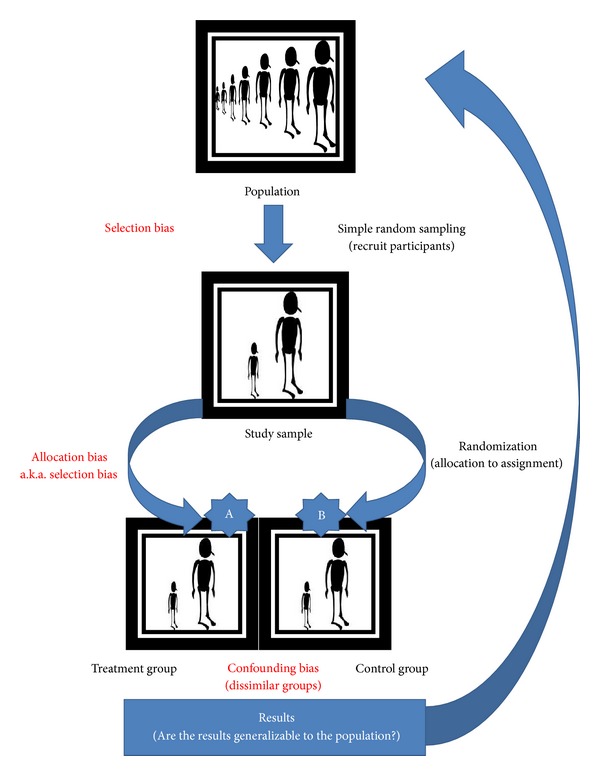
Generalizability using simple random sampling.

Therefore, it is difficult to think how we can achieve generalizability using simple random sampling when conducting purposive sampling selection often used in experimental clinical and social science research (Figure 1). That is, if external validity can be in play at all, what good is our inference of the causal relationship to hold over variation in persons, settings, treatment variables, and measurement variables based on a singular random sample from a population. Shadish et al. [9] believe that external validity is variations in persons, settings, treatments, and outcomes that *were* in the experiment and for persons, settings, treatments, and outcomes that *were not* in the experiment. They treat generalization of causal relationships from a single sample to unobserved instances as a matter of external validity—whether or not random sampling was used. We would argue that without measurement of external validity in trials (both efficacy and effectiveness studies) we risk failing to translate research into public health practice [16].

#### 1.3.3. The Selection by Eligibility Criteria: Confounding Factors and Attrition Bias

A confounding factor (also known as confounding variable) is an extraneous variable in a statistical model that correlates (positively or negatively) with both the dependent variable and the independent variable. These factors include age, gender, educational levels, risk factor, life style, and environment. These factors often have impact on health status and so should be controlled. Attrition bias is a type of selection bias. Attrition bias arises when some groups of people withdraw (or are withdrawn due to adverse events or lack of adherence to study protocol) from the research study more frequently than others; in turn the sample no longer resembles the original sample in the study.

The selection criteria also known as the inclusion and exclusion criteria are important to reducing both confounding variables (internal validity) and attrition bias (external validity) for research studies. However, in systematic review methodology, it is very important to tease apart the influences of variations in internal and external validity on effect size estimates [22]. Often RCTs have well-defined inclusion and exclusion criteria. Narrowing the eligibility criteria for patients usually means stronger internal validity and increases overall effect size for specific patient populations [22]. However, patient characteristics such as age, gender, severity of disease, and risk factors influence the total effect size at both levels, internal and external validity. A study can have a strong internal validity but weak external validity at the population level. We will use an example illustrated by Persaud and Mamdani [18] of spironolactone use in heart failure which illustrates the danger in relying on specific selection criteria that is internally but not externally valid.

“The Randomized Spironolactone Evaluation Study (RALES) was prematurely ended when its conclusion became clear: “Blockade of aldosterone receptors by spironolactone, in addition to standard therapy, substantially reduces the risk of both morbidity and death among patients with severe heart failure” [23]. But, compared with the RALES, a retrospective study found a significantly higher incidence of hyperkalemia when spironolactone was combined with standard therapy [24]. Further, a population time-series analysis showed that the increase in spironolactone prescription among elderly congestive heart failure (CHF) patients after the RALES was followed by an increase in hyperkalemia-related hospitalizations and hyperkalemia-related hospital deaths for this patient group [25].

The differences between the findings of the RCT and the non-experimental studies are thought to be due to the application of findings from a group of patients with a particular feature (i.e. low risk of hyperkalemia) to actual patient populations. The RALES suggested that spironolactone can reduce mortality in CHF patients in combination with standard treatment if used in selected patients under careful observation, while the non-experimental studies suggested that in actual clinical practice spironolactone does not reduce mortality at the population level. In short, the application of “level one” evidence to clinical practice required re-interpretation in the light of so-called “inferior evidence” from non-experimental studies [18].”

The previous example illustrates how specific eligibility criteria can produce strong positive effects in select patients but at the population level cannot produce those effects and/or creates adverse events. Therefore, systematic reviews should account for the representative of patients remaining in the study due to (1) inclusion and exclusion criteria, (2) dropout and withdraws, (3) adverse events, and (4) lack of adherence to treatment protocol. It is important to address baseline prognosis in subgroups that were under-represented in RCTs (i.e., elderly, women, blacks, etc.) or in the general population of patients outside of the specialized centers where trials are most often completed [18].

Lastly, to increase internal validity, many RCTs exclude the participation of patients with comorbidities. However, these same studies (which excluded a subsample of patients) provide justification of evidence for use on intervention or treatment for all patients (including the excluded sub-sample of patients). The exclusion of patients with comorbidities from RCTs can lead to external validity bias and potentially inadequate and/or dangerous approach to treatment [26].

## 2. Methods

A computerized database search, PubMed (including MEDLINE), EBM (Evidence-Based Medicine) Reviews-Cochrane Central Register of Controlled Trials (CCTR), and Cochrane Library was performed from database inception up to January 2, 2013, to identify relevant articles published in the English language, describing or using an external or model validity tool or checklist to evaluate the methodological quality of RCTs in health care research. Key words used in the search strategy included “external validity,” “model validity,” “bias-scoring” plus “scale,” “checklist,” “critical appraisal,” “critical appraisal review,” “appraisal of methodology,” “research design review,” “quality assessment,” and “randomized controlled trial.”

**Figure 2:**
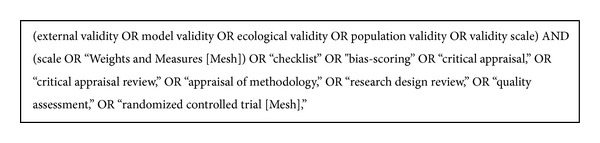
Search terms used according to MeSH strategy.

The search terms used according to our MeSH strategy is outlined in Figure 2.

Published studies reporting on scale development or the psychometric evaluation of an external validity and model validity scale, checklist [27] or domain-based evaluations such as the Cochrane Collaboration's tool for assessing risk of bias [4] were eligible for inclusion.

## 3. Results

The initial electronic database search of the literature resulted in a total of 1131 article abstracts. Of these 33 were selected as potential studies based on their title and abstract. In addition to the findings of Moher and colleagues [1, 2] and Olivo and colleagues [3] we identified 8 additional scales, checklists, or domain-based evaluations that include external validity and/or model validity assessment for RCTs. These tools include (1) the Effective Public Health Practice Project Quality Assessment Tool (EPHPP) [28]; (2) the Berhoft et al. checklist for model validity and external validity qualitative evaluation of clinical studies [29]; (3) the Mathie et al. checklist for model validity and external validity qualitative evaluation of clinical studies [30]; (4) the Dekkers et al. strategy to assess the external validity and applicability of clinical trials [11]; (5) GAP (assessment of generalizability, applicability and predictability) for evaluating an external validity checklist [21]; (6) the Downs and Black checklist for validity [31]; (7) LOVE [20]; and (8) Singh Scale [32].

### 3.1. Tool Development

We have developed an instrument for assessing the external validity of both RCTs and non-randomized studies in health care interventions. This tool was adapted using the literature pool collated from the tools identified with the criteria most essential for determining external validity of study results and following the suggestions of Cochrane Collaboration Risk of Bias assessment approach [8] and Scottish Intercollegiate Guidelines Network approach for guideline methodology [7]. In addition, we used two checklists (the GAP checklist [21] and the Downs and Black checklist for* measuring study quality*) [31] especially from the pooled tools identified, to refine our external validity and model validity dimensions when building our external validity assessment tool (EVAT).

We assessed the feasibility of EVAT using a consensus approach. Two or more reviewers (RK, CC, JS) individually and independently assessed the methodological quality of the external and model validity dimensions of RCTs and effectiveness studies using EVAT, with a rulebook developed by the authors, and refined the criteria and objectivity of the tool based on their assessments until consensus was reached. The rulebook, which allows for an objective measurement while minimizing reviewer bias, will be used to train the Samueli Institute internal core systematic review team in the use of EVAT to further test the validity and reliability of the tool in all upcoming systematic reviews.

### 3.2. EVAT: External Validity and Model Validity Formalized in a Bias Scale

External validity assessment tool (EVAT) methodology is encouraged to be useful for each clinical health care study, in assessing its external and model validity, ensuring authors report on these criteria so that systematic reviews can evaluate them for their strengths of external and model validity. The methodology assessment is based on three core domains that focus on external and model validity aspects of the study design. Our literature review of evidence found particular aspects of the study design that have a significant effect on the risk of bias in the study results and conclusion. We used our knowledge gained through the literature review described previously and the overarching prevalent themes through the tools already documented (especially the GAP [21] and the Downs and Black [31] checklist) to come up with this objective, streamlined tool.

EVAT is created for methodological rigor in addition to practicality of use (see Table 1). For more information on EVAT and accompanying note on its use is available through contacting the Samueli Institute (http://www.samueliinstitute.org/). Understanding this tool and its applicability is useful for clinical trialists to ensure they are reporting on these criteria in all reports which then can be assessed as quality criteria by systematic review methodologists.

EVAT methodology is based on three core domains. Each of the domains is assessed based on the reporting of each individual study. The reviewer is placed to consider each of the domains based on the aspects of the study design and make a decision as to how well that individual study meets that domain criterion. For each domain, one of the following is chosen to indicate quality addressed in each study: (1) well covered (++), (2) adequately addressed (+), (3) poorly addressed (−), and (4) not applicable (0).

### 3.3. Domain 1: The Study Addresses Study Recruitment

The first domain is on the identification of the source population for participants as reported by clinical trials, both RCTs and non-randomized studies. That is, were the participants recruited from that source population (i.e., identify the source population for participants and describe how the participants were selected)?

### 3.4. Domain 2: The Study Addresses Participation

Unless a clear description of the study sample is specified based on the representative of the entire source population, it will be difficult to assess how well the study has demonstrated that the distribution for any relevant risk factors and mediating or confounding variables has an effect on the study conclusions. It is important to note that this domain question is only applicable if Domain 1 was answered, either well covered or adequately addressed. If marked poorly addressed, this question would also have to be marked poorly addressed because the reviewer would not have an understanding of the source population as described in the report.

### 3.5. Domain 3: The Study Addresses Model Validity

This domain addresses the concept of model validity. It is important that there is a clear and detailed description of staff, setting, and intervention characteristics that would enable judgment of relevance to other settings representative of the treatment the majority of patients receive.

Inevitably, the assessment process in systematic review methodology involves some degree of subjectivity made by the individual reviewer. That is, an individual reviewer brings the research/clinical context as well as personal judgment to the appraisable process. Therefore, the EVAT methodology, to reduce and to minimize personal bias or subjective errors, requires that each study selected for the full review consideration be appraised by at least 2 or more reviewers. If there is a disagreement on the EVAT domain between 2 or more reviewers, a third reviewer (i.e., primary investigator, program manager, etc.) will lead as the arbitrator and consensus must be made before the study is included in the systematic review evidence base. Samueli Institute has made the attempt to create the tool to be more objective and the evaluation more streamlined by creating a rulebook on how to choose the different categories of answering the criteria.

## 4. Discussion

### 4.1. External Validity and Model Validity in Clinical Decision Making and Its Applicability

For clinical decision making external validity includes the generalizability of the intervention to the actual patient population. In 2009, Jonas and colleagues [33] study to assess the external validity of RCTs published in four primary care journals by quantifying the selection process of trial enrollees from the primary care population found that the reporting of recruitment to RCTs based in primary care is inconsistent and frequently incomplete. They state:

“Of the 148 RCTs published, 103 (70%) reported the number of individuals who were screened for eligibility and 80% of published RCTs reported the number of individuals who were eligible. In those RCTs that did report on the recruitment process there appears to be marked variation in terms of the proportion of individuals who are recruited. These findings suggest that reporting of RCTs should be improved and that for some RCTs external validity is limited because only a low proportion of eligible participants are successfully recruited [33].”

Therefore, those RCTs that lack recruitment data need to be treated with caution as it may represent inadequate identification or reporting of the eligible population. In the future, systematic reviews should consider the recruitment data when evaluating external validity.

As stated previously, differing views on conceptual systems of etiology, diagnosis, and pathophysiology itself lead to different types of study designs [20]. A recent study by Bell and colleagues [34] on the methodological implications of nonlinear dynamical systems models for whole systems of CAM (WSCAM) states that:

“In research terms, “good” randomized clinical trial (RCT) designs have strong internal validity to test efficacy; that is, well-controlled experimental conditions are chosen to optimize certainty that the independent variable alone (i.e., a specific intervention) produced the observed effect. External validity, on the other hand, addresses the generalizability of an observed effect for the larger population from whom the study participants were drawn; and model validity relates to the concordance between the study design and an idealized setting (for more details, see [29, 35]). For external and model validity in studies, WS-CAM practitioners would need to (a) use indivisible, interdependent, iterative packages of care rather than isolated standardized elements of a package; (b) ensure that practitioners involved in studies are well trained and highly experienced; (c) treat real-world patients with confounding co-morbid conditions, psychosocial factors, and other treatments; and (d) evaluate complex, emergent patient-wide outcomes in each case, rather than the simple organ- or disease-specific effects. The major features of WSCAM include coordinated, adaptive, and indivisible packages of care and emergent positive patient-wide outcomes in both global function (e.g., quality of life) and multiple local subsystems (e.g., “non-specific” changes in many symptoms, not just the original chief complaint). Taken together, these clinical complexities are fundamentally incompatible with the assumptions of conventional biomedical efficacy research study designs for standardized interventions to assess specific outcomes [34].”

Fundamental differences of the worldviews of the reductionistic biomedical model compared to the holistic conceptual model for CAM/IM create complex methodological problems. These methodological issues include problems with general linear model assumptions used by conventional research methods, including RCTS. Linear and reductionist theory assumptions include the following: (1) proportional cause and effect relationship; (2) causes are independent; and (3) residual errors are a sample of independently and identically distributed [34]. As stated above, CAM/IM research may not fit well into the linear research model and may require a complex nonlinear model. Therefore, CAM/IM RCT statistic conclusions, or lack thereof, should be interpreted with caution. Pragmatic quasi experimental design may be better suited for CAM/IM interventions and treatments. By including EVAT in reporting of clinical trials as well as the evaluation of quality in systematic reviews, we can interpret the results to be more generalizable in future studies to make better informed decisions about health care.

In 2012, Fernandez-Hermida and colleagues [21] reported that of the 29 included RCTs in their study, the majority (69%) did not report sufficient information for judging generalizability from sample to study population, 35% did not report sufficient information for judging applicability to other populations and settings, and no study provided predictability based on an assessment of the validity of the trial end-point measures for subsequent mortality, morbidity, quality of life, or other economic or social outcomes. In addition, not a single study reported on the validity of surrogate measures using established criteria for assessing surrogate end-points. Therefore, they concluded that studies evaluating the benefits of family-based prevention of alcohol misuse in young people are overall inadequate at reporting information relevant to generalizability of the findings for health or social outcomes. In fact, it has been discussed that many RCTs may not be particularly useful, relevant, or noteworthy, even when they are conducted meticulously, in part because these trials are designed to estimate the impact of an intervention “under *ideal* circumstances in which the intervention is most likely to show benefit” [36]. Therefore these trials are often called ‘‘explanatory trials,” or “efficacy studies” and may only be useful for answering questions about whether an intervention can work under ideal circumstances but the results are of limited relevance to answering questions about whether an intervention does work under practical or usual circumstances, and therefore are less useful to clinicians, managers, and policy makers [36]. Others have discussed how much of modern biomedical research is operating in areas with very low pre- and poststudy probability for true findings. Ioannidis states, “most new discoveries will continue to stem from hypothesis generating research with low or very low pre-study odds. We should then acknowledge that statistical significance testing in the report of a single study gives only a partial picture, without knowing how much testing has been done outside the report and in the relevant field at large” [37].

For all of research, no matter what type of study design is intended to be carried out, a clear research question with adequate information relevant to generalizability of the findings must be asked first and foremost. Some trials are more focused on efficacy where other trials, the main goal is to test the effectiveness of an intervention. Efficacy refers to the extent to which a specific intervention is beneficial under ideal conditions whereas effectiveness measures the extent to which a specific intervention, when deployed in the field in routine circumstances, does what it is intended to do for a population. Both types of studies can be conducted using the RCT study design. However, efficacy and effectiveness studies are very different in their approach to their research questions, focusing on different aspects of validity. As one can imagine, an efficacy study would score higher on internal validity components where an effectiveness study relates more to the external validity components assessed and would score lower on internal validity and higher on external validity components. Depending on the clear research question defined and the objective of the study, a pendulum is created when one introduces internal validity and external validity criteria to be evaluated. Effectiveness RCT study designs suffer in systematic review methodology as they are currently mostly evaluated using internal validity criteria by the pure nature of the research question being evaluated. Offering a tool that evaluates both internal validity and external validity will allow a reviewer to see this pendulum swing and ensure that the researcher is addressing the most important criteria for answering the specific research question in a comprehensive rigorous way. The purpose of research is to serve the audience intended to create change. Several audience members exist such as patients, providers, the community, and policy decision makers. These audiences are interested in some common core information pieces but with differing priorities. We as researchers need to find a balance and agreement of what the overlapping information required to help these members to begin to make decisions for health care. The Patient-Centered Outcomes Research Institute (PCORI) was developed to conduct research to provide information about the best available evidence to help patients and their health care providers make more informed decisions. The goal of this initiative is to give patients and those who care for them a better understanding of the prevention, treatment, and care options available to them and the science that supports those options.

The amount of quality research available on CAM/IM practices is still quite small compared with that on conventional medicine. For much of conventional medicine, efficacy studies seem to make the most sense where there is a very precise research question that can be answered with the standard RCT for drug studies. For CAM/IM, however, some of these systems are quite complex and without the necessary requirements for reporting of these trials, it becomes a challenge to generalize the results. For example, practices like acupuncture require considerable training and skill to be properly delivered. There are many different licensing structures in the United States and abroad and without fully disclosing the level of training or certification experience in the report, it is challenging to generalize the results to other populations. More complex assessment tools that are sensitive to these areas are needed.

EVAT was created to fill a gap for assessing external and model validity for the Samueli Institute Rapid Evidence Assessment of the Literature (REAL©) [38] methodology. EVAT is a rapid assessment tool that will be added in future studies planned at the Samueli Institute through upcoming systematic reviews. A proprietary rulebook for answering these questions has been developed to train our internal team of reviewers and will be used in the forthcoming systematic reviews. The psychometrics of EVAT will be tested in upcoming systematic reviews and the gaps on not reporting these real-life application criteria will emerge.

## 5. Conclusions

In conclusion, due to failure to measure external validity and model validity, practitioners are often unable to determine if a given study's findings apply to their local setting, population staffing, or resources. The lack of information on external validity and model validity can contribute to the failure to translate research into public health practice.

Therefore, policy and administrative decision makers are unable to determine the generalizability or breadth of applicability of research findings. Finally, systematic reviews and meta-analyses are limited in the conclusions that can be drawn when external validity data are not reported. EVAT offers researchers a way to account for these complexities when evaluating research and the validity of the research to apply to real-world utility. With proper training in the use of this assessment tool, we can have a more robust, rigorous assessment of clinical trials that will better serve those making clinical decisions for their patients and communities.

## Figures and Tables

**Table 1:** EVAT* for assessing the external validity of both RCTs and non-randomized studies in health care interventions.

	Well Covered (++)	Adequately Addressed (+)	Poorly Addressed (−)
(1) *Recruitment* Did the study identify the source population for participants and describe how the participants were recruited from that source population?			
(2) Participation** Were those subjects who participated in the study representative of the entire source population from which they were recruited?			
(3) *Model validity* Were the staff, places, and facilities where the patients were treated representative of the treatment that the majority of patients would typically receive?			

*EVAT is a modified tool based on the GAP checklist and the Downs and black checklist for measuring study quality.

**This question is only applicable if question number 1 was answered, either well covered or adequately addressed. If marked poorly addressed, this question would also have to be marked poorly addressed because the reviewer would not have an understanding of the source population as described in the report.
